# Editorial Comment: Portable model for vasectomy reversal training

**DOI:** 10.1590/S1677-5538.IBJU.2019.0092.1

**Published:** 2019-01-29

**Authors:** Rodrigo R. Vieiralves

**Affiliations:** 1 Hospital Federal da Lagoa Rio de Janeiro RJ Brasil Serviço de Urologia, Hospital Federal da Lagoa, Rio de Janeiro, RJ, Brasil

We know that microsurgery training is still far from becoming a reality during the process of training residents in Brazil. In fact, there is a gap around the world and therefore, just the fact that we have an article dealing with this topic is already of great relevance. In this very interesting article conducted at Para State University, Brazil (1), a portable training model for vasectomy reversal was adopted. We know that with the current juncture of society and the number of vasectomies performed worldwide, there is an increasing demand for vasectomy reversal mainly associated with a new post-vasectomy relationship. The paper in question becomes even more relevant because of it´s bimodal certification for microsurgical training based on a time performance and on a check list not only measuring the execution time of vasovasostomy but also with a checklist questionnaire assessing the most diverse specific items of an perfect anastomosis (eg, if the resident is placed in a comfortable position, equidistance between knots, number of knots, continuous needle vision, etc). We understand that this bimodal evaluation increases the accuracy in the training, since the time does not necessarily correlate with the quality of the anastomosis.

However, some relevant aspects need to be highlighted. Vasectomy reversal surgery involves a complex number of factors for its true success. The preparation of the surgical field itself, the section of adhesions and previous fibrosis, the calibration and approximation of the deferens ends without tension, the stabilization of the anastomosis, the patency test, the observation of the four deferent layers in the intraoperative period -mucosa, two layers of muscle and the adventitia- (failure to observe two muscle layers may indicate residual vasectomy scarring). Blood supply evaluation by thin mucosal bleeding is also important (2). None of these fundamental points for surgical success is reproduced through the present model.

Moreover, we know that the technique used in the model, proposed by Benlloch (3) with only 4 sutures in a single layer is not a reference in the literature. Today, we found no statistical difference in patency or pregnancy results for two- and one-layer vasovasostomy but a minimum number of 6 sutures presumably offers a high quality anastomosis by preventing sperm leakage and the associated risk of granuloma (4, 5).

Some considerations regarding the materials used should be made. A technical point is the fact that using an 8-0 suture could facilitate training without simulating real anastomosis situations. We understand that further in vivo studies with thicker sutures in a single anastomotic layer are needed to validate this model (6, 7). Also, as much as the 3D printed model addresses important aspects such as the presence of two layers allowing the training of two types of anastomoses, single layer or double layer, the consistency of the material hardly simulates the vas deferens real physical proprieties, since the external PVA coating probably offers a much higher resistance than the real one. Other point is that there is no exact description of how the model is fixed on a training table, since any mobilization makes all training difficult and inaccurate. The magnification used while using the microscope has not been described also.

Regarding the validation model, the low number of residents who perform the training draws attention. We understand that results based on training of only 5 residents can present an important bias, due to individual aspects besides the fact that they are part of the same Hospital (similar previous training), making the external validation of this model difficult. Another point about this model is the aspect of 5 different days of training. Although undeniably a quality model, we understand that a final assessment 8-12 weeks after the last training session would be important for permanent/long term skill acquisition assessment. This is of great relevance as we know that vasectomy reversal and microsurgery will not be a routine urologist procedure.

To conclude, we see in this important paper the presentation of a vasectomy reversal training model that is reproducible, storable, transportable, low cost and durable. It allows a single or double layer training, and despite the need for improvement in the validation model, we undoubtedly have a non-animal 3D model that sets a benchmark for future improvements, serving as a basis for more realistic ones. It will be possibly used as the first stage of basic training in microsurgery and vasectomy reversal.
